# Osteogenesis Imperfecta: A study of the patient journey in 13 European countries

**DOI:** 10.1186/s13023-024-03345-0

**Published:** 2024-09-09

**Authors:** Ingunn Westerheim, Valerie Cormier-Daire, Scott Gilbert, Sean O’Malley, Richard Keen

**Affiliations:** 1 Osteogenesis Imperfecta Federation Europe (OIFE), Schotelveldstraat 17, Heffen, 2801 Belgium; 2 Reference Center for Skeletal Dysplasia, Paris Cité University, INSERM UMR 1163, Imagine Institute, Hôpital Necker-Enfants Malades, 149 rue de Sévres, Paris, 75015 France; 3 Putnam Associates, 22–24 Torrington Place Fitzrovia, London, WC1E 7HJ UK; 4 Royal National Orthopaedic Hospital NHS Trust, Brockley Hill, Stanmore, Middlesex HA7 4LP UK

## Abstract

**Introduction:**

Osteogenesis imperfecta (OI) is a heritable skeletal disorder and comprises various subtypes that differ in clinical presentation, with Type I considered the least severe and Types III/IV the most severe forms. The study aim was to understand the OI patient diagnostic and treatment journey across Europe.

**Methods:**

We conducted a qualitative, descriptive study to understand the OI patient journey. A selection of people with OI/their caregivers and clinicians involved in OI-patient care from across Europe were interviewed using a specially developed questionnaire.

**Results:**

Between May 2022 and July 2022, 22 people with OI/caregivers and 22 clinicians (endocrinologists, orthopaedic surgeons, geneticists and metabolic specialists) from across Europe were interviewed. Our study showed various areas of concerns for the OI community. Timely diagnosis of OI is essential; misdiagnoses and a delay to treatment initiation are all too common. There are a lack of consensus guidelines regarding optimal treatments (including when bisphosphonate therapy should be initiated and the route of administration) and patient management throughout the duration of the patient’s life. Adult OI patients do not have a medical home and are often managed by endocrinologists and rheumatologists. Adult care is often reactive based on the development of new symptoms. The psychosocial burden of OI impacts on the patient’s quality of life.

**Conclusions:**

There is an urgent need for increased awareness about OI and its wide range of symptoms. In particular, there is a need for consensus guidelines outlining the optimum care throughout the duration of the OI patient’s life.

**Supplementary Information:**

The online version contains supplementary material available at 10.1186/s13023-024-03345-0.

## Introduction

Osteogenesis imperfecta (OI) is a heritable skeletal disorder, which is caused by the defective formation of bone [1]. OI is a complex condition that is attributed to numerous genetic mutations affecting more than 20 different genes, as outlined in the 2023 revision of the nosology of genetic skeletal disorders. [2, 3] For example, mutations in the collagen 1 genes – COL1A1 and COL1A2 – account for approximately 90% of all cases [4, 5]. OI is usually classified according to clinical type based on severity, but many different genetic mutations can occur within one clinical type. As a result, it is more common to divide patients according to clinical type/severity (e.g., Sillence types I–V) and include additional information on specific genetic mutations and their mode of inheritance [6]. Many individuals with OI can have significant physical disabilities (either visible or invisible to the outside observer) and the degree of disability varies according with severity [7]. Type I OI, the most common type, is associated with blue sclera and childhood fractures (although some patients may not experience fractures until later in life). Type II OI is usually described as lethal in the pre- and perinatal period, although other definitions also exist. Type III OI is the most severe form that is compatible with survival, with patients suffering from numerous fractures, severely short stature, bowed long tubular bones, and scoliosis of the spine. Type IV OI has a varied presentation and associated with moderate deformation [8, 9]. These four main types account for up to 90% of all OI types. [10] Other genetic types of OI also exist, [9, 11] but are outside the scope of this manuscript. OI is a rare disease under the EU and US definitions [12, 13] and, based on data from Sweden, has a prevalence of 5.16, 0.89 and 1.35/100,000 population for OI Types I, III and IV, respectively (overall prevalence 7.40/100,000 population) [8]. 

Although bisphosphonates are widely used off-label, particularly in children and for the treatment of people with OI who are at risk of fractures, there is currently no cure for OI and there are no therapies approved by the EU or the US regulatory authorities for this condition. Optimal management of OI would involve a multidisciplinary team (MDT) providing an array of services including occupational therapy, physiotherapy, orthotics, psychologists, dieticians, orthopaedic surgeons and those clinical specialists involved in the provision of pharmacological support (e.g., the administration of bisphosphonates and pain relief) [14]. Expectations of people with OI and clinician aspirations for the outcome of OI management vary according to OI type and disease severity, but the overarching goal is for with people with OI to achieve active and independent lives that are less influenced by their condition [15]. 

The aim of our study is to gain a clearer understanding of the diagnostic and treatment journey for OI from the perspective of people with OI, their caregivers and their clinicians, and to determine the challenges faced by people with OI and their families along this journey.

## Methods

### Design

This is a qualitative, descriptive study designed to understand the OI diagnostic and treatment journey from both the perspective of people with OI and treating clinicians. Research was conducted by a third-party agency and is outlined below.

### Data sources

People with OI, their caregivers and treating clinicians from across Europe (Belgium, Finland, France, Germany, Italy, Netherlands, Norway, Poland, Spain, Sweden and the United Kingdom) were included in this analysis. The ‘OI group’ included people with OI and their caregivers. OI group participants were identified through the Osteogenesis Imperfecta Federation Europe (OIFE). All individuals completed a pre-interview screener, which confirmed a diagnosis of OI, prior to participating in the research and provided informed consent for inclusion in the study. The clinician group included healthcare professionals who are actively engaged in the treatment and clinical research of OI, both from a paediatric and adult perspective, including endocrinologists, metabolic specialists, geneticists, orthopaedic surgeons and rheumatologists. Clinicians were identified and recruited by partners including Atheneum, Guidepoint and Techspert who specialise in the recruitment of experts in rare diseases in the EU. Potential clinician interviewees completed a pre-interview screener, which captured information on their specialty, country of practice, volume of OI patients treated, OI publication history and clinical trial participation. The aim was to recruit 20 subjects for both the OI and clinician groups, respectively.

### Interview questionnaires

Interview questionnaires were developed through peer-reviewed publication review using PubMed search term: osteogenesis imperfecta treatment and selecting five publications. Interview questionnaires comprised four main areas – patient evaluation and diagnosis, referral and MDT, paediatric treatment, and transition to adulthood. The questionnaires contained 20 questions for the clinicians compared with 24 questions for the OI group (see Appendix I). Interviews were conducted Zoom from a central location in either the United States or the United Kingdom by a specialist third-party agency with extensive experience in interviewing patients, carers and clinicians on the topic of rare diseases. Each interview lasted approximately 60 min. Participants in the OI group had the option to choose whether the interview was conducted with or without a video; HCP interviews were conducted without the use of videos. The first interview was conducted on 25 May 2022 the final interview was conducted on 25 July 2022. All interviews were recorded and pseudonymised patient data was stored in a secure, password-protected database. Upon completion of the interviews, any knowledge gaps in the diagnostic and treatment journey were supplemented through literature searches.

### Data analysis

The data was reviewed and interpreted by a team of three analysts from the specialist third-party company. Analysis included a review of the interview transcripts and populating the data in an Excel-based data capture file to ensure consistency across each respondent. Responses were then categorized into relevant themes. Analysts used this information to identify themes and/or experiences that arose most commonly across the respondent set.

## Results

### Interview subjects

Overall, 44 people in the OI group and clinicians were identified as potential interview subjects. Between 25 May 2022 and 25 July 2022, 22 people with OI/caregivers and 22 clinicians from Europe were interviewed.

Clinicians from Belgium (*n* = 2), France (*n* = 3), Germany (*n* = 3), Italy (*n* = 3), the Netherlands (*n* = 1), Poland (*n* = 1), Spain (*n* = 3), Sweden (*n* = 3) and the UK (*n* = 3) were interviewed. Overall, the clinician group comprised adult endocrinologists (*n* = 4), paediatric endocrinologists (*n* = 3), adult orthopaedic surgeons (*n* = 1), paediatric orthopaedic surgeons (*n* = 2), adult rheumatologists (*n* = 4), paediatric rheumatologists (*n* = 2), clinical geneticists (*n* = 2) and metabolic specialists (*n* = 3). Interviewee demographics are presented in Table 1. The annual exposure of clinicians to people with OI ranged from 21 to 88 patients/year (data not shown).

**Table 1:** Clinician group participant demographics

	Clinician group
Country	
Belgium France Germany Italy Netherlands Poland Spain Sweden UK	2 3 3 3 1 1 3 3 3
Specialty	
Endocrinologist - Adult Endocrinologist - Paediatric Orthopaedic surgeon - Adult Orthopaedic surgeon - Paediatric Rheumatologist - Adult Rheumatologist - Paediatric Clinical geneticist Metabolic specialist	4 3 1 2 4 2 2 3

Interviews were conducted in 22 subjects in the OI group (16 patients and 6 caregivers) from Belgium (*n* = 2), Finland (*n* = 1), France (*n* = 2), Germany (*n* = 2), Italy (*n* = 3), the Netherlands (*n* = 1), Norway (*n* = 1), Poland (*n* = 2), Spain (*n* = 3), Sweden (*n* = 2), and the UK (*n* = 2). The clinical types of OI reported by the patient group included Type I (*n* = 4), Type III (*n* = 8), Type IV (*n* = 5) and unknown (*n* = 5). There were no cases of Type II OI reported, likely due to extreme symptoms and mortality in infancy. Interviewee demographics are presented in Table 2.

**Table 2:** OI group participant demographics

Subject	OI group (clinical)	Pt age	Age at Dx	Time to Dx^†^	Diagnostician	Misdiagnosis before OI diagnosis	Visit to CoE	Country of residence
1 (C)	III*	17	Prenatal	24 weeks	Endocrinologist	N	Y	Belgium
2 (Pt)	IV	58	Birth	15 mo	Orthopaedic surgeon	Y; soft bones	N/A	UK
3 (Pt)	U	35	~ 1 y	~ 1 y	Paediatrician	Y; NAI, malnutrition	Y	Italy
4 (Pt)	I	37	Birth	Birth	U	Y; NAI	Not for adults	Sweden
5 (Pt)	IV	23	Few mo	Few mo	U	N	U	Germany
6 (Pt)	III	41	After birth	U	U	N	Y	Norway
7 (Pt)	III*	35	After birth	Birth	Orthopaedic surgeon	U	Y	Netherlands
8 (Pt)	I*	29	Prenatal	Prenatal	OI bone specialist	N	Y	UK
9 (Pt)	U	28	~ 10 y	3–4 y	Neurologist^‡^	Y; malnutrition	Y; not for adults	Poland
10 (C)	IV*	3	1 y	1 y	Orthopaedic surgeon	Y; NAI	Y	Sweden
11 (Pt)	U*	53	12–15 y	U	Geneticist	Y; “Rochstein” disease^§^	Y	France
12 (Pt)	III	35	Birth	Birth	Paediatrician/GP	N	N; too far	Belgium
13 (Pt)	U*	29	2.5 y	U	Orthopaedic surgeon	Y; NAI	Y	France
14 (C)	U*	12	4 mo	4 mo	Radiologist	Y; NAI	Y	Italy
15 (C)	III*	9	2–3 mo	2–3 mo	Geneticist	Y; clavicle fracture	Y	Spain
16 (C)	III*	6	Birth	Birth	Paediatrician	N	Y	Spain
17 (C)	I*	8	Birth	Birth	U	U	Y	Italy
18 (Pt)	IV*	46	~ 1 y	9–18 mo	U	N	Y	Poland
19 (Pt)	IV*	53	6 months	Few hours	Orthopaedic surgeon	N	N	Spain
20 (Pt/C)	III	34	Prenatal	Birth	U	N	Y; adults and children	Finland
21 (Pt)	I	54	~ 12 y	2 mo	U	Y; NAI	Y	Germany
22 (Pt)	III	23	Birth	Birth	Specialist	N	Y	Spain

*Genetic testing conducted to confirm IO diagnosis and, in some cases, the patient’s OI subtype and results given to patient/family. ^†^Time to diagnosis was based on patients describing their experiences. Slight discrepancies in time to diagnosis, particularly for patients 2 and 18 where the time to diagnosis exceeds the patient’s age at diagnosis, are likely due to the patient’s recall of exact timings/events. ^‡^Family member. ^§^This represents the patient’s description of their misdiagnosis

Where C, carer; CoE, centre of excellence; Dx, diagnosis; GP, general practitioner; mo, months; N, no; N/A, not applicable; NAI, non-accidental injury; P, pharmaceutical treatment; Pt, patient; S, surgical treatment; U, unknown; UK, United Kingdom; y, year; Y, yes

### Diagnosis of OI

#### Clinician perspective

**Fig. 1:**
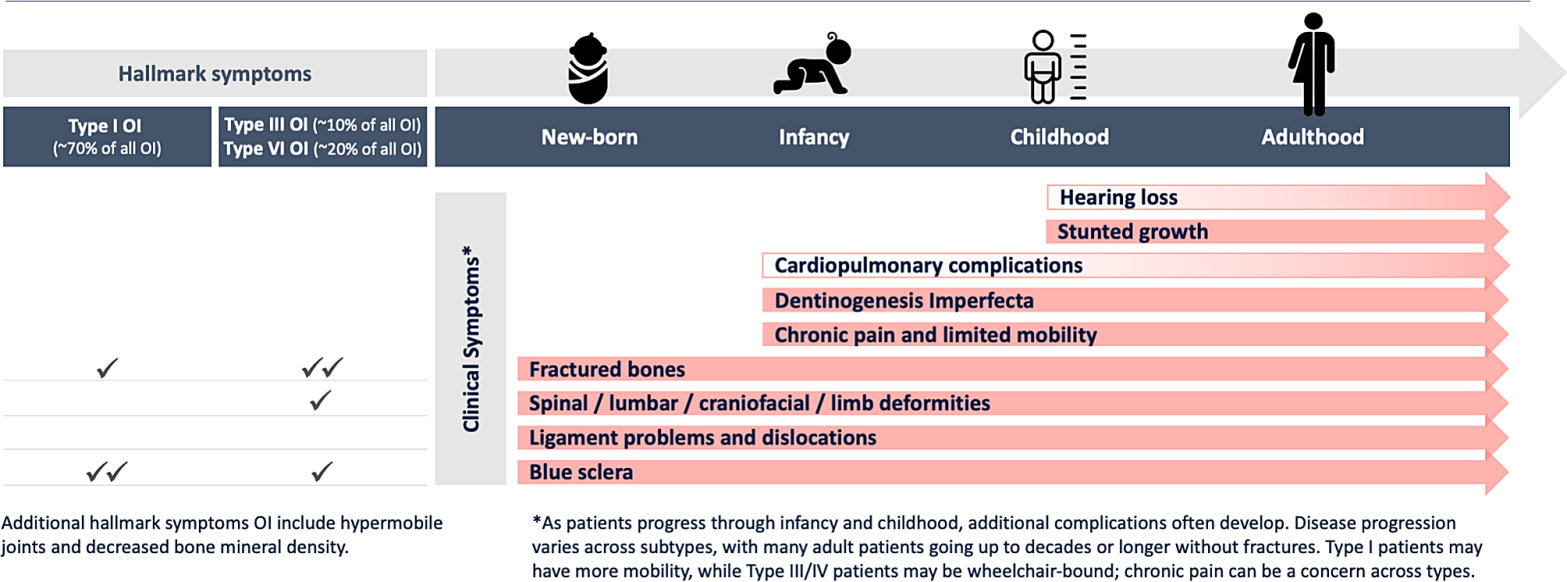
The most common symptoms of OI by stage of development*. *Legend* OI, osteogenesis imperfecta

People with Type I OI typically comprise ~ 70% of the overall OI population. The most common symptoms that trigger an OI evaluation in Type I OI patients included blue sclera, fractures, hypermobile joints and decreased bone mineral density (Fig. 1). These patients were typically diagnosed between 3 and 5 years of age when they had a greater ability to move around independently with more frequent falls leading to bone fractures; often patients have a family history of OI. People with this type of OI typically presented with unusual or unexplained fractures in early childhood to primary care physicians (PCPs), paediatricians or emergency room (ER) clinicians or, less commonly, to dentists with discoloured, crowded and/or weakened teeth. Less severe cases, however, may not be identified until adolescence or even adulthood.

People with Types III and IV, which together account for ~ 30% of all OI cases, were usually diagnosed *in utero* or shortly after birth. They typically had either a family history of OI or short/bowing of the long bones either detected in an ultrasound during pregnancy or at birth by obstetricians/gynaecologists or neonatalists. Presentation included fractures, decreased bone mineral density, hypermobile joints, deformities, and distinctive craniofacial features. They may also have had pale blue sclera at birth but this typically fades during the first year of life. Regardless of the type of OI, as patients progressed through infancy and childhood, additional complications often developed (Fig. 1), but disease progression was impacted by OI type.

Once a suspicion of OI was raised, regardless of the route, patients underwent various diagnostic tests to confirm an OI diagnosis including DEXA scans, radiographical imaging, genetic testing (multigene panels including COL1A1 and COL1A2) and/or blood tests to rule out other diagnoses. The availability of genetic testing varied across Europe, but when it was not available in-house most centres made referrals for testing.

#### OI group perspective

From the OI group perspective, the diagnostic journey for people with OI is less well-defined. For those people who are diagnosed in early life, their journey frequently started off in an emergency setting due to the presence of unexplained bone fractures. In rare incidences it raised the concern of non-accidental injury (NAI/child abuse). For example, one person stated that “*They took my child because they thought I’d beaten her”*, this was particularly evident where the parents appear healthy/have no history of OI and the child is in substantial pain due to having broken bones. An NAI allegation could be traumatic and psychologically damaging for the family. Other misdiagnoses included Ricketts, Ehlers-Danlos Syndrome, juvenile osteoporosis and hypophosphatasia. Hypophosphatasia is of particular concern because the use of bisphosphonates – a staple treatment for OI – is contraindicated in these patients.

Another concern was a delayed diagnosis, particularly in those people with less severe OI symptoms. This was attributed to a lack of OI awareness among paediatricians and/or general practitioners (GPs) and the variability in severity and presentation of symptoms. One endocrinologist suggested that *“People who are Type I with more mild clinical symptoms may take longer before making a diagnosis because their symptoms are very non-specific.”* A delayed diagnosis could delay treatment and corrective action, resulting in worsening health in the longer term.

### Care of OI patients: paediatric patients

#### Clinician perspective

**Fig. 2:**
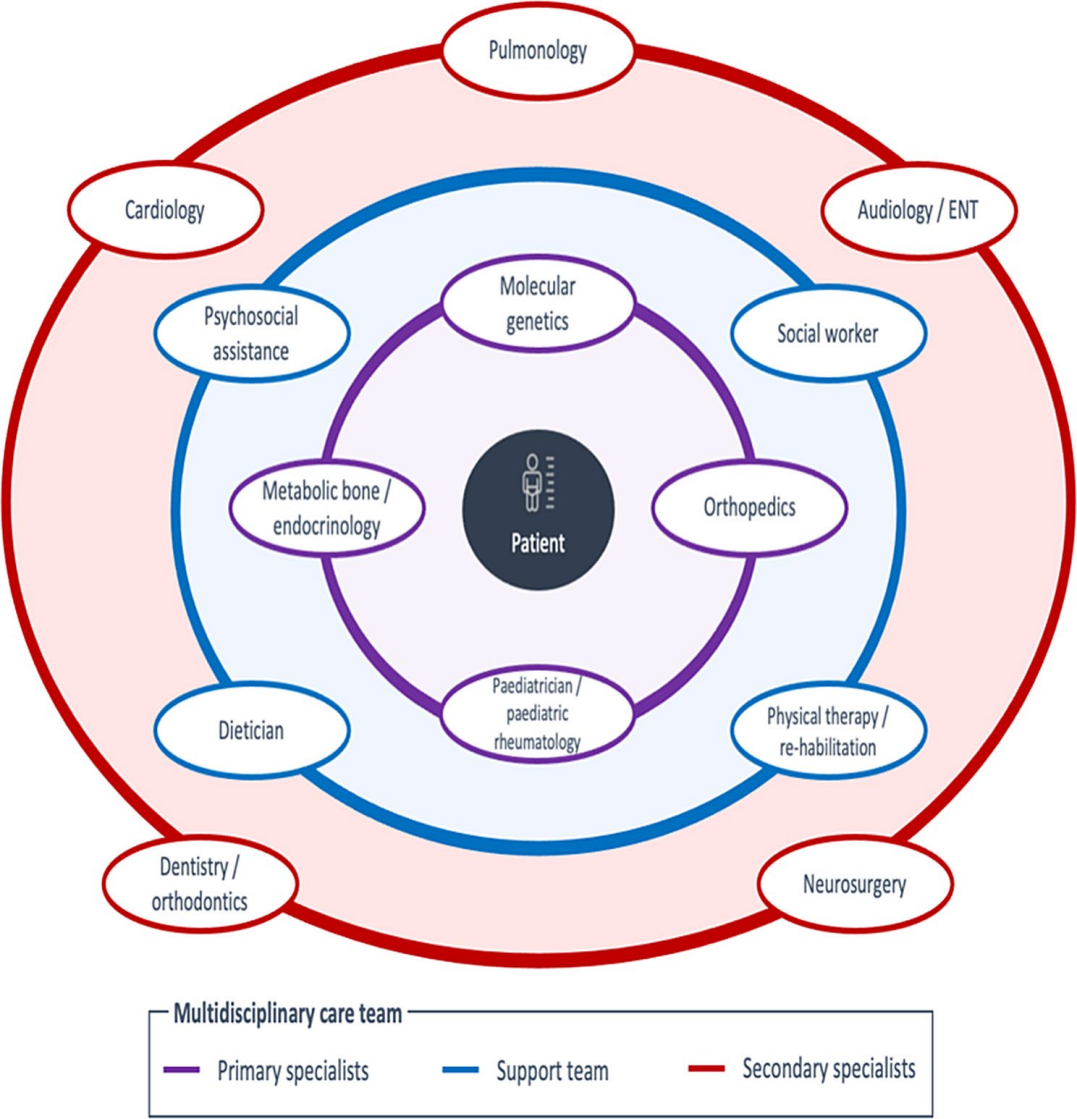
OI multidisciplinary care team. *Legend* ENT, Ear, nose and throat specialist

The involvement of different clinicians in the management of OI is presented in Fig. 2. Briefly, the primary specialists included geneticists to assist with the OI diagnosis, orthopaedic surgeons for corrective surgeries/fracture management, paediatricians/paediatric rheumatologists and metabolic bone specialists/endocrinologists who served as the medical home for OI patients and managed the use of pharmacological treatments (e.g., prescription of bisphosphonates). The support team for people with OI varied according to country but could include psychosocial assistance, social workers, physical therapists/rehabilitation specialists and dieticians. For example, of the countries interviewed, only France, Germany, Spain and the UK had access to psychologists in their MDTs at centres of excellence (CoE); in Sweden people with OI could be referred to psychologists through their CoE. Finally, secondary specialists were recruited as needed according to the patient’s OI symptoms and could include cardiologists to monitor for age-related risks of heart failure, atrial fibrillation and valvulopathies (although these complications are generally perceived as a greater concern for adult patients than in children); pulmonologists; audiologists/ENT specialists for hearing loss; neurosurgeons; and dentists/orthodontics for dentinogenesis imperfecta. In an ideal scenario, people with OI would access their treatment through CoEs, however, this varied across Europe. France appeared to have the most advanced referral network, with a high clinician awareness of OI and a patient-friendly database of expert care centres (OSCAR) that is maintained by the French rare bone disease organisation (a Department of Health initiative). Other countries, such as Belgium, referred people with OI to university hospitals in absence of CoEs, but these countries were under pressure from patient advocacy groups to designate CoEs.

The key treatments for people with OI included bisphosphonate therapy administered by paediatric endocrinologists, metabolic bone specialists or paediatric rheumatologists, which was usually discontinued upon growth plate closure, fracture cessation or the end of adolescence (see Table 3 for an overview of the different treatments received by each patient). Most clinicians used either the number of annual fractures (≥ 2 fractures/year) or bone mineral density as a threshold to initiate bisphosphonate treatment. The most common bisphosphonates were pamidronate administered over 1–3 consecutive days at 2–4-month intervals and zoledronic acid, a higher potency alternative with a 30-minute infusion time typically administered every 6 months.

**Table 3:** Overview of patient treatment

Subject	OI group (clinical)	Pharmacological intervention	Surgical intervention	Other interventions
1 (C)	III*	Pamidronate from age of 5 months; switched to zoledronate at ~ 12 years	Rodding and straightening	Rehabilitation/PT
2 (Pt)	IV	Alendronate, but stopped treatment due to side effects	Traction and rodding	PT in adulthood, hearing aids and dentures
3 (Pt)	U	Neridrontate and ibuprofen	Rodding	PT and wheelchair
4 (Pt)	I	Pamidronate	Nails in knee for broken bones; nails kept coming out so removed	Calcium supplements and foot supports for back pain
5 (Pt)	IV	Bisphosphonates	Rodding and nails; nails were later removed	Calcium supplements and rehabilitation/PT
6 (Pt)	III	Too old for treatment when bisphosphonates became SoC	N/A	Wheelchair, hearing aids, and breathing aid while sleeping
7 (Pt)	III*	As a child, bisphosphonates	Rodding (arms and legs); spinal fusion	Vitamin D, calcium, PT, wheelchair
8 (Pt)	I*	Started on risedronate, switched to zoledronate; part of the setrusumab trial	Screws for broken ankle, spinal surgery for scoliosis, stapedectomy for hearing issues	PT, hydrotherapy, manual wheelchair, walking frame and hearing aids
9 (Pt)	U	Not qualified for treatment	Rodding in legs, surgeries to address fractures	PT, wheelchair
10 (C)	IV*	N/A	Rodding	Vitamin D, calcium supplements and walker (post-surgery)
11 (Pt)	U*	N/A	Fracture repair with nails	Hearing aids
12 (Pt)	III	Pamidronate (from 14–21 years)	Fracture repair	Vitamin D, wheelchair, hearing aids and CPAP machine (night time only)
13 (Pt)	U*	Bisphosphonates	Fracture repair	PT, rocking chair for long periods of sitting, orthopaedic brace, Chinese medicine
14 (C)	U*	Neridronate	Rodding (both legs)	Hydrotherapy, wheelchair and walking. Considering getting a wheelchair-friendly car
15 (C)	III*	Started on pamidronate; switched to zoledronate	Rodding (arms and legs)	PT, swimming, wheelchair and braces
16 (C)	III*	Started on pamidronate, switched to zoledronate; denosumab	Rodding (arm) and fracture repair (other arm)	Calcium supplements, vitamin D, PT, wheelchair and hearing aids
17 (C)	I*	Neridronate and acetaminophen	Arm fracture repair and rodding	Vitamin D and PT
18 (Pt)	IV*	N/A	Bone curvature correction	PT, wheelchair and crutches (as needed)
19 (Pt)	IV*	Started on pamidronate, switched to zoledronate; pain medication	Nails to correct bone curvature	PT, swimming, home workouts, wheelchair
20 (Pt/C)	III	Part of a clinical trial (medication not specified)	Leg deformity corrections	PT, swimming, wheelchair
21 (Pt)	I	Ibandronate	Nails and fracture repair	Rehabilitation
22 (Pt)	III	Started on pamidronate, switched to zoledronate	Rodding (legs) and hip replacement	Calcium supplements, vitamin D, PT, walker and crutches

*Genetic testing conducted to confirm OI diagnosis and, in some cases, the patient’s OI subtype and results given to patient/family

Where C, carer; CPAP, continuous positive airway pressure; N/A, not applicable; Pt, patient; PT, physical therapy; SoC, standard of care

Orthopaedic surgeons provided fracture repair; rodding to address limb bowing and deformities; revision, correction or extension surgeries; and spinal fusion surgery to address scoliosis and other spinal deformities. The surgical burden for people with OI can range from 2 to 3 surgeries in a lifetime for people with Type I OI to > 50 surgeries (comprising rod placement, extension and correction accounting for the majority of surgeries) for people with Type III/IV OI. For people with dentinogenesis imperfecta, the dentist or orthodontist provided orthodontic treatment, tooth extraction and dentures as needed.

#### OI group perspective

Most people with OI considered it challenging to be seen by OI specialists, with one person stating *“It was really*,* really difficult to get seen by a specialist*,* so I had to become a bit of an expert in how the* [country-specific healthcare provision] *works”*. However, as children, most would visit a CoE that focuses on rare diseases, rare bone disease or OI specifically. In some European regions, where there were either low densities of OI experts or concentrated specialised care (e.g., Belgium, Poland and Sweden) it was common for people with OI to travel long distances to access specialist care. This resulted in a significant travel burden for some families, with one person commenting that *“We went to* [CofE] *– 3 hours to go and 3 hours to return. They say you can go in a day*,* but it is impossible.”*, which meant that interaction with specialists occurred only once or twice yearly.

As previously stated, the key treatments for OI included the use of bisphosphonates and surgical interventions. Some of the surgical treatments included dental repair, fracture repair, spinal fusion and rodding. While these may be considered ‘standard’ procedures for people with OI; the impact of these procedures is significant. One person with Type IV OI explained their experience of tooth extraction *“My earliest memories are being held down to get a tooth extracted. Now there’s more awareness of the risks of extractions – they may cause some fractures in jaws and things. And now I just more or less have full dentures”*. Other people provided information on their surgeries *“The spinal fusion surgery was a lot harder*,* that took more time. I think that might have been 3–6 months and also with intensive physical therapy afterwards.”*; and *“The recovery went well. But the only thing is there was a very big cast – from the breast to the leg. It was a big challenge for us.”*.

### Transition to adult care and ongoing management

Around the age of 18, most people with OI usually transition from paediatric to adult care. There is no established standard of care for adults with OI. While the majority of adult OI patients are not followed by specialised healthcare teams, some countries – France, the Netherlands, Sweden and the UK – generally continued to monitor OI progression in adults, but with a limited MDT. These patients tended to be monitored by adult endocrinologists or rheumatologists. Adult patients reported feeling frustrated and overwhelmed by the need to identify their own ‘team’ of doctors from a limited pool of experienced specialists. Patients become disillusioned by one-dimensional care from non-experts in OI who may only address the immediate issue rather than looking at the disease in greater context. In addition, many patients adapt to reduced fracture risks and do not see the need to seek care until other age-related symptoms develop (e.g., hearing loss, chronic pain and stress fractures).

The majority of adult patients are not treated pharmacologically due to a negligible fracture rate, lack of available treatment options and/or issues with access to care. Some patients who continue to experience fractures may continue with IV bisphosphonate treatment (e.g., pamidronate, zoledronic acid and neridronate) or switch to oral bisphosphonates (alendronate, risendronate and ibandronate). Furthermore, clinicians noted that oral bisphosphonates have only a modest impact at best on bone strength, fracture rate and pain in adult patients but they may be prescribed in absence of other effective treatments. This may result in treatment compliance concerns, with only a minority of adults either restarting or continuing treatment. Furthermore, most clinicians wanted to see at least 2–3 fractures/year before prescribing treatment which, in some countries, was linked to reimbursement. However, most cases of less severe adult OI will never reach this threshold.

Clinicians were aware of other pharmacological agents that may be beneficial for OI patients, such as denosumab and teriparatide, but their use was limited.

### Psychosocial burden

There is a significant psychosocial burden for people with OI. Upon entering adolescence and adulthood, people with OI generally become more comfortable with managing their condition but the psychosocial symptoms had a larger toll on their quality of life. People with OI often adopted a cautious approach to everyday activities because of the constant threat of fractures. One caregiver explained that *“We are constantly wired to see the risk in every situation. We make sure he doesn’t climb anywhere. It’s just precautions always. And he knows it too*,* he doesn’t take as much risk. Even when he sits down*,* he does it in a certain way.”*. Furthermore, accessibility for OI-related disabilities (e.g., mobility, stature and hearing) lead to a feeling of social exclusion. This could be overcome by OI associations providing a group of peers with shared experiences. The challenges also extended to healthcare scenarios. At CoEs, clinicians are more familiar with OI; outside of these settings patients felt that their condition was poorly understood and that their concerns were either trivialised or ignored. One person with Type IV OI commented that *“I became aware that menopause can be a particularly add-on effect. The Brittle Bone Society were recommending that you have a DEXA scan as a control before you went into menopause and when I asked my GP about that*,* he just laughed”*. Another area of concern was around family planning, where genetic counselling was needed for patients to understand the scope of their options.

### Additional support for people with OI

Oftentimes, people with OI will turn to patient advocacy groups for additional support. Most countries in Western Europe had OI groups, but these were less prevalent in Eastern European countries. Some OI groups function as a helpful source of community and resources for OI, others also aided in referrals to CoEs or assisted with CoE transparency. Patient experiences of OI representative groups varied across Europe. In France, one patient said *“When I went to a conference*,* there was like super men and women. They were doing major things with their bodies. And I was like ‘OK*,* if they can do it*,* maybe I can too’. It was really inspiring.”* while the experience in Poland was different *“We have a small organisation. But there is no brainstorming about cure or something; no new ideas how to help each other. It’s like just to meet*,* drink and socialise”*.

## Discussion

To the best of our knowledge, this is the first study to comprehensively review the OI patient journey from diagnosis to ongoing management from a European perspective involving interviews with both clinicians and people with OI and their caregivers alike. From this study, it is clear that these two groups have different perspectives at each stage of the journey. For clinicians, there are specific timepoints for the diagnosis of OI – typically *in utero* or immediately after birth for patients with Type III/IV OI and in early childhood for patients with Type I OI. All patients in our study were diagnosed with OI by the age of 15 years. The overarching treatment and management goal is for with people with OI to achieve active and independent lives that are less influenced by their condition. One of the pain points for clinicians is lack of disease awareness in the general medical setting and the best practices for treating people with OI. Clinicians would benefit from guidelines for managing OI patients – particularly on the preferred pharmacological interventions and their duration of use.

The journey to diagnosis for people directly affected by OI is often traumatic – a lack of disease awareness can result in misdiagnosis (including NAI) and a delay in starting treatment, which might impact on the severity of the disease pathway. Even after diagnosis, the challenges remain – in accessing specialist care/CoEs (including navigating the referral system via general practitioners and travelling to the CoE), undergoing complex surgical procedures that require long rehabilitation times, understanding the duration of pharmacological treatments, transitioning to adult care, sourcing experts to continue with proactive treatment and disease management and the psychosocial burden of OI.

OI is a rare disease with a heterogenous presentation. Our manuscript looked at the differences between clinical Type I and Types III/IV OI. Clinical Type I OI is often considered to be a ‘mild’ form of the disease [9]. However, it is important to remember that clinical manifestations of OI are not defined by disease type or even the affected gene, but depend on many different factors including the location and type of gene mutation(s) [16]. Patients with Type I OI can suffer from multiple and repeated bone fractures from minor impacts, [17] blue sclera, dentinogenesis imperfecta, hyperlaxity of ligaments and skin, hearing impairment, marginally shorter stature and bone deformities [11]. While people with Type I OI have a normal lifespan, they still require considerable support from the medical community to ensure optimum health.

Previous studies have investigated the impact of living with OI and the socioeconomic impact of the disease [18, 19]. In line with our study, these showed that symptoms, diagnostic techniques and the use of pharmacotherapy in children were well-documented and that there is an increased need for disease knowledge. However, less was known about the current care practice, the diagnostic journey, patient monitoring and the impact of OI on the patient’s wellbeing and quality of life. The findings from our study have provided some of this much needed information.

The biggest problem with OI is that it is a rare disease. Rare diseases are associated with various challenges for patients, clinicians and researchers alike. For people living with rare diseases, the challenges centre around obtaining a diagnosis, receiving optimal care, finding appropriate specialists/accessing knowledgeable general health service providers, a lack of evidence around effective treatments and, for some conditions, accessing affordable disease-specific medications. Conversely, obstacles for clinicians include gaining both knowledge and experience in managing patients with rare diseases, the availability of local experts and lack of expert guidelines, while researchers have problems finding sufficient numbers of subjects for studies and accessing funding [20, 21]. There are an estimated 7,000 rare diseases, with up to 80% of these being related to genetics [22, 23]. Due to their low incidence, rare diseases are infrequently discussed in medical education programmes and are often difficult to diagnose [24, 25]. All of these challenges hold true for OI and broadly correlate with the findings of our study. Different geographical regions have adopted various strategies to improve the patient diagnostic and treatment journey for those with rare diseases, with further recommendations being raised, such as building capacity and awareness amongst healthcare workers, supporting research and development, and building public awareness [26]. In addition, various countries have also adopted a fast-track review process for newly developed treatments for rare diseases [27, 28]. 

For those patients who are fortunate to obtain a rare disease diagnosis, the challenges remain as they seek access to the best management and care [29]. For OI, like many rare diseases, there is low awareness of the condition outside of specialist centres and there are no clinical or best practice guidelines to support patients and clinicians alike to achieving a timely diagnosis or to define lifelong patient management. The European Reference Network on rare bone diseases (ERN BOND) was established to improve access to high-quality healthcare for patients with rare bone diseases, such as OI, and is in the process of developing guidelines for this patient population. Even with the development of guidelines and the promotion of adoption strategies, however, it can take years before they are implemented in clinical practice [30]. Guidelines for OI are much needed to harmonise patient management and to improve ways of working within this disease area.

The need to increase disease awareness of OI is huge and it affects many stages of the patient journey. Fractures are considered to be one of the most common signs of NAI/child abuse – occurring in an estimated 50% of maltreated children [31]. In our study, six families received a misdiagnosis of abuse prior to their OI diagnosis. Differentiating between OI and suspected child abuse is challenging for clinicians due to an overlap in symptoms, and misdiagnoses have occurred all too frequently [32]. For families with OI this can result in an inherent mistrust of the medical profession. Raising awareness of OI isn’t only necessary within the medical profession. OI impacts numerous aspects of the patient’s life including safety concerns when planning for holidays, the spatial layout of the home and accessibility for wheelchair users, choosing a school and participating in social activities, especially those outdoors [33]. A greater understanding and awareness of OI would help to alleviate some of these pressures.

Bisphosphonates, used to increase bone mineral density and prevent fractures, are one of the most commonly prescribed treatments for OI, and many patients in our study received this treatment [34]. Interviews with clinicians in our study also confirmed the use of bisphosphonates for OI patients, but highlighted that there is currently no consensus on when bisphosphonate therapy should be initiated, the preferred method of administration (intravenous vs. oral therapy for paediatric and adult patients) or the duration of therapy. Treatment is typically initiated in childhood but often stops after growth plate closure. Furthermore, data is needed on the long-term fracture reduction and, even more so, on the other, non-fracture-related impacts of OI; as well as how long term use of bisphosphonates impacts on quality of life [35]. Although collecting this data will be challenging because it will require natural history studies or the use of registries with regular and consistent follow-up of people with OI, once it becomes available it needs to be incorporated into consensus guidelines to promote the optimised care of OI patients. In addition to mainstay treatments, further information on the use of up-and-coming potential OI treatments is needed, such as denosumab and teriparatide, and the use of experimental treatment strategies. Our study confirmed that, in addition to pharmacological intervention and surgery, our patients often received other interventions. More information is also needed on the routine use of these other supportive therapies for OI, such as vitamin D therapy, the use of pain treatments and access to mental health support. Despite the availability of treatments to manage the symptoms of OI, there is no cure for this disease.

The patient journey for children with OI is not the same as for adults and patients with rare diseases need complete care throughout their lives. This is compounded by a lack of natural history data for OI. A particular pain point for people with rare diseases is the transition from paediatric care to adult care [36–38]. Our study showed that this is also an area of concern for people with OI. Oftentimes there were no adult OI specialists for patients and their care was managed by adult endocrinologists or rheumatologists. Care is also reactive with patients visiting their general practitioners and asking for referrals to specialists as symptoms or concerns manifest. The development and implementation of guidelines would help to support this transition pathway.

Patients are also becoming increasingly resourceful about researching their illnesses and making connections with other patients, either directly or through the OI representative groups. These groups, predominantly motivated by patient need, are increasingly being found at the forefront of policymaking, research and drug development for their disease of interest [39]. Our study has showed that such groups for OI exist across Europe and that they play a vital role for people with OI and their caregivers, from helping to identify CoEs to providing expert advice. Cohesion of the OI representative groups across Europe may also promote enhanced OI care.

Overall, the findings from our study have highlighted the complex and heterogenous journey for patients with OI from diagnosis, disease management, transition to adult care through to the impact of disease burden and need for additional lifestyle support. It is hoped that the findings of this study will raise awareness of OI as a rare disease among members of the medical community, help expedite the development of treatment guidelines and show the urgent need for therapeutics to help support and, ultimately, cure this disease.

### Study limitations

The study was designed to include a wide range of subjects from across Europe. However, only a small handful of subjects were interviewed in each country. Due to the heterogeneity of OI, it is possible that the interviewees may not have had all the symptoms that are associated with OI and some information may have been missed. Furthermore, the interviews provided a wealth of information on the diagnostic and treatment journey for patients with OI, but there is always more information that could have been gleaned to make our understanding of the diagnostic journey more robust, such as the approximate number of clinician appointments before a diagnosis was achieved. In addition, chronic pain is a common experience for patients with OI, [40] it would be interesting to understand how this impacts on the patient journey to diagnosis, as well as how pain is managed. It is also well-recognised that people with OI treated at expert centres will have a different experience to those treated in a community setting. It would have been interesting to capture what proportion of people with OI interviewed were supported by expert centres and community settings because this has the potential to influence the data. Finally, the data captured in these interviews is from people with OI, families, carers and clinicians based in Europe. While a wide range of countries were included in this study, it is possible that the findings may be less applicable to other geographical regions, particularly those with less developed and/or poorly funded healthcare provision.

## Conclusions

To the best of our knowledge, this is the most comprehensive review of the diagnostic and treatment journey for patients with OI in Europe. To make this study as complete as possible, information was obtained from interviews with both people with OI and clinicians, and supplemented through literature searches to fill knowledge gaps. This study shows how heterogenous OI is as a disease but also how varied the diagnosis and management of people with OI is across Europe. Guidelines and/or expert consensus reports are notoriously difficult to develop for rare diseases due to the small numbers of patients but would be of enormous benefit for OI. In the absence of established treatments for OI, guidelines would provide invaluable support in improving an approach to management and monitoring of OI patients that continues from the paediatric setting through to adulthood.

## Electronic supplementary material

Below is the link to the electronic supplementary material.

Supplementary Material 1

## Data Availability

The datasets for this study will not be made publicly available due to data protection reasons but are available from the corresponding author on reasonable request.
