# Supplementary material for: Osteogenesis Imperfecta: A study of the patient journey in 13 European countries
Source: Orphanet J Rare Dis. 2024 Sep 9;19:331. doi: 10.1186/s13023-024-03345-0 (PMC11386111; doi:10.1186/s13023-024-03345-0)
Supplement: Supplementary file 1 — Supplementary Material 1 [file 13023_2024_3345_MOESM1_ESM.docx]

**APPENDIX I**

**Patient questionnaire**

1. Please tell us a little about yourself.
2. How old are you/your loved one?
3. How long have you/your loved one had symptoms of OI?
4. How would you describe your/your loved one’s overall health?
5. In addition to OI, do you/your loved one have any other chronic diseases or health issues?
6. **[For respondents with OI]** How would you describe your lifestyle?
7. How active are you?
8. What day-to-day activities are most important to you?
9. Do you work outside the home? If so, what is your occupation?
10. **[For respondents with OI]** Do you have a family member or caregiver who helps you manage your OI?
    1. **[If yes]** How involved would you say this caregiver is in helping you manage OI? How involved is this caregiver in learning about treatment options and discussing your treatment plan with your doctor?
    2. **[If involved]** What are some examples of how this person helps you? Does he or she accompany you to your doctors’ appointments? Ask questions of the doctor? Joined a support group or spoken with other caregivers?
    3. Are there other people not including your doctor or nurse who help you manage OI?
    4. How has OI impacted your and your caregiver’s/family’s lifestyle?
11. **[For both respondents with OI and caregivers]** When it comes to dealing with OI, what are the issues that are most important to you? **[Probe on importance of minimizing fracture risk, managing pain, maintaining ability to work or participate in social/family activities]**
12. **Symptom Onset, Diagnosis, AND REFERRAL**
13. **[For respondents with OI and caregivers]** What are your early memories of the disease?
    1. When did you/your family first realize your loved one/you had OI?
    2. Which OI subtype was diagnosed? Were you made aware of the specific OI subtype at diagnosis?
    3. Who explained what OI is to you and how?
    4. Did you learn about OI from any sources other than your HCP?
14. **[For caregivers]** What were the symptoms of that first made you seek medical attention for your loved one?
15. Who first noticed these symptoms? **[Probe on person with OI, caregiver, doctor]**
16. How long did you observe these symptoms before you sought medical attention?
17. Were there any specific symptoms that caused a specialist to suspect OI at this point?
18. **[For caregivers]** Which types of doctors or specialists were involved when you were trying to figure out what the different symptoms meant?
    1. Were there multiple doctors involved? How were their roles different from each other? **[Probe on analyzing specific symptoms (e.g., multiple fractures, limited mobility, bone deformity, etc.) and the exact sequence of specialties involved, including rationale]**
    2. How long was the entire process from the emergence of the first symptoms to formal diagnosis? **[Probe on delays / wait time for specialist referral]**
19. **[For respondents with OI and caregivers]** Who ultimately made the OI diagnosis?
    1. Do you remember which different diagnostic tests were performed to help identify the condition?
       1. Was genetic testing used to confirm the diagnosis? **[Probe on whether diagnosis occurred using pre-natal genetic testing]**
    2. Which specialty finally made the diagnosis of OI? Is this the same doctor that manages routine care for OI?
    3. What was the sequence of referrals among different physicians that brought you to the specialist who eventually made the OI diagnosis? **[Probe on the number of specialists seen prior to diagnosis, type of specialists seen, where these specialists were located etc.]**
       1. At any point were you referred to a specialist or center far from where you live? By whom? Why?
       2. Were there any stages or hurdles in the referrals where you felt held up or “lost”? Why? **[Probe on specialists not realizing the underlying cause of the condition, worsening of symptoms, etc.]**
20. **[For both respondents with OI and caregivers]** Was there ever misdiagnosis with another condition before the correct diagnosis of OI? **[If so:]**
21. Do you remember which condition OI was first misdiagnosed as?
22. Do you know what caused doctors to suspect diseases other than OI? **[Probe on specific symptoms leading to misdiagnosis/delayed diagnosis, and which doctors made the misdiagnoses]**
23. If OI was diagnosed as another disease, how long did it take for the doctor to realize this and re-diagnose as OI?
24. **[For both respondents with OI and caregivers]** How did you feel when you received the diagnosis?
25. How was OI explained to you? Did the doctor explain what type of care or treatment might be necessary?
26. Knowing what you know now, is there anything you would do differently related to the diagnosis process?
27. **[For both respondents with OI and caregivers]** How did the disease impact your / your loved one’s life during infancy, childhood, adolescence, and transitioning to adulthood?
    1. What is your life like now? How has it changed since you were younger?
    2. How do you think OI shaped you and the way you lead your life?
    3. How did you / your loved one’s OI symptoms progress with age?
       1. What are some of the most common complications or medical symptoms you have faced during the different stages of the disease?
       2. Which ones have been the most difficult for you?
28. **[For both respondents with OI and caregivers]** Have any of your / your loved one’s siblings or relatives been diagnosed with OI?
    1. Does having relatives with OI make managing the disease easier? Harder? Why?

1. **[For both respondents with OI and caregivers]** What group of doctors do you seek when you/ your loved one need/needs urgent help?
   1. In your opinion, who is the primary decision maker for management of OI?
   2. What are the roles of the other specialists in management of OI?
   3. What do you/your loved one do in emergency situations in the absence of specialty physicians or in atypical situations where you / your loved one are not near your / their local treatment center?
2. **Current Treatment and Management**
3. Which doctor(s) or specialist(s) do you currently for treatment and management of OI?
   1. Is there a team of specialists or doctors that manages treatment?
      1. What specialists are a part of this team?
      2. Who is the most involved with your/your loved one’s management?
      3. Is there anyone else, besides doctors, involved in managing your treatment? **[Probe on role of care coordinator, if any]**
   2. Were there any specific centers you/your loved one visited for the treatment?
   3. How frequently do you see doctors for treatment of OI?
4. **[For both respondents with OI and caregivers]** What treatments or procedures have you / your loved one used or undergone to treat OI? **[Probe around pharmacological standard of care and surgical interventions]**
   1. Are you currently using any drugs to treat OI? Which ones? **[Probe on use of bisphosphonates, PTH analogues]**
      1. What did your doctor tell you about this drug before treatment began?
      2. Are you satisfied with this treatment? What symptoms of your disease does it help with most?
      3. Have fractures occurred while on treatment? How frequently?
      4. Since starting treatment, have you ever suspended treatment for any reason? **[Probe on voluntary drug holidays vs. inability to procure or access drug]**
      5. **[If used in the past, but not currently]** Why did you discontinue treatment with this drug? Do you use something else instead now to manage OI?
   2. Have you / your loved one undergone any surgeries to treat OI? **[If yes:]**
      1. How many surgeries have you / your loved one had? Over what period of time?
      2. How many different types of surgeons have you / your loved one seen?
      3. What was the reason for these surgeries? Were they helpful in alleviating symptoms or complications of OI?
      4. What was the recovery process from these surgeries like?
   3. Have you / your loved one ever needed rehabilitation or physical therapy as part of treatment for OI?
      1. Why did you need rehabilitation or physical therapy?
      2. How long did you use rehab services?
   4. Have you had any challenges affording or obtaining insurance coverage for any of these treatments or surgeries? **[Probes will vary by market]**
      1. **[If yes]** Which ones? How did you manage through these challenges?
      2. Do you have access to any subsidies or support to offset any costs of OI treatment or associated travel costs? From where?
      3. Were there any treatments or surgeries that were recommended to you but that you didn’t receive because you couldn’t afford them or because they weren’t covered? **[Probe on if patients ever suspended treatment for this reason]**
5. **[For both respondents with OI and caregivers]** How did your/your loved one’s treatment for OI evolve with time? When do you remember first receiving it?
6. How did your/your loved one’s treatment progress or change over time?
   - 1. What were the changes? Did the treatment/management change drastically at any point? If so, how did you/your loved one and you handle it?
7. What symptoms are you most focused on managing today? **[Probe around recurrent fractures, pain management]**
8. **[For adults with OI]** Did the treatment and management of OI change as you transitioned from pediatric to adolescent to adult care?
   1. Was there a formal transition plan or process?
      1. Did you see the same doctor(s) as a child that you see now? If not, why? Were there doctors who would no longer see you past a certain age?
      2. Do you receive treatment at the same center that you did as a child, or did you have to find a new treatment center? If so, how did you find a new treatment center? **[Probe on role of patient advocacy or support groups]**
   2. How frequently do you see your doctor(s) for treatment or follow-up visits? How did the frequency change from when you were a child?
   3. Did you experience any specific challenges related to your treatment and disease management during the transition to adulthood?
9. **[For both respondents with OI and caregivers]** What are some of the biggest challenges or “pain points” you / your loved one have had in terms of managing OI treatment?
   1. Looking back at your experience with OI, what do you think were the hardest parts of the experience to manage? The most inconvenient? **[Probe on lack of effective treatments, inconsistency of outcomes, pain management, challenge of coordinating care across specialists]**
   2. How does managing OI impact your day-to-day life? **[Probe on psychosocial and economic impact of disease and treatment, impact from missed school / work, travel expenses for treatment and surgeries, etc.]**
   3. Is there anything you would have done differently related to your / your loved one’s treatment of OI knowing what you know now?
10. **UNMET NEEDS IN OI**
11. **[For both respondents with OI and caregivers]** What would you consider to be some of the biggest unmet needs or things that you would like to change with the management of OI?
    1. What are the most important areas where you would like to see changes at each point in your journey with OI? **[Probe on lack of effective therapies, access to surgical interventions and mobility aides, difficulty coordinating care, diagnostics at each developmental milestone and transition between points of care]**
    2. Which of these specific areas are the highest burden to you or your family?
12. **[For both respondents with OI and caregivers]** What were some of the biggest challenges you experienced with the treatment options the doctor offered you/your loved one?
13. Was there anything specific you/your loved one experienced that you felt the doctors were able to manage well with therapies? **[Probe on the management of the secondary comorbidities experienced]**
14. Were there symptoms that you/your loved one felt the doctors were not able to offer anything to manage?
15. Which of these was most frustrating?
16. **[For both respondents with OI and caregivers]** How satisfied are you with the current management options available to treat OI symptoms?
17. Are there any key symptoms that require better treatment options more than others?
18. How does the lack of an OI treatment that can change the course of the disease impact you/your loved one’s and your family’s quality of life? **[Probe on the social, psychological, and economical impact on quality of life, level of anxiety over fractures]**
19. **[For both respondents with OI and caregivers]** At any time, have you/your loved one been involved in any patient support groups or patient advocacy groups related to OI?
20. **[If yes]** Which one(s)? How did you learn about them?
21. What role do these networks play in your lives? How have they benefitted you and your loved one? **[Probe on role in finding specialist care]**
22. In what areas could you use more support? What type of support would help the most?
23. Have you ever participated in a clinical trial or considered participating in one? Why or why not?
    - 1. If so, how did you become aware of this clinical trial?

1. **[For both respondents with OI and caregivers]** What would you most want in a new treatment for OI? **[Probe on ability to reduce rate of fractures, side effects, symptom management]**

a. Have you heard of any new drugs in development? Where did you hear about these?

b. How could a new drug potentially improve the management of OI and alleviate some of the challenges you previously mentioned?

c. What would be the most impactful to your quality of life?

1. **Conclusion**
2. Is there anything that we have not yet discussed during today’s discussion related to the management of OI that you feel we should cover?

**Clinician questionnaire**

1. Please provide a brief overview of your background and role within your practice. **[Confirm screener responses]**
   1. Practice setting (academic vs. community)
   2. Current role, including leadership responsibilities
   3. Involvement in research
   4. Time spent in direct patient care
2. Please describe your typical role in the diagnosis and management of patients with osteogenesis imperfecta (OI). **[Confirm screener responses]**
   1. How many OI patients you have managed in the last 12 months?
3. **Symptom Onset, Diagnosis, and Referral (15 min)**
4. What are the typical initial signs and symptoms of OI that lead patients and caregivers to seek medical attention?
   1. When do the signs and symptoms first appear? Do they tend to occur in any particular order?
   2. Who first notices these signs and symptoms? **[Probe on patient, caregiver, type of HCP]**
   3. How long do patients typically experience signs and symptoms before patients/caregivers seek medical attention or an HCP expresses concern?
   4. Are there any key signs and symptoms that may cause an HCP to suspect OI at this initial presentation stage?
   5. How often are key signs and symptoms of OI confused with other diseases? Which other diseases are most commonly suspected or misdiagnosed?
   6. How does patient presentation differ across the subtypes of OI? **[Probe on most common signs and symptoms and age at presentation]**
      1. Beyond OI subtype, what other segments or patient characteristics are meaningful as it pertains to initial presentation **[Probe on age and frequency / onset of fractures]**
5. How long does it typically take from when a patient first presents with signs and symptoms to when they are diagnosed with OI?
   1. How old is the patient, on average, at the time of diagnosis?
      1. How much does this vary by subtype?
   2. Do you think the time between onset of signs and symptoms to diagnosis with OI could potentially be shortened? If so, how?
      1. What typically causes this delay? **[Probe on lack of awareness of OI by initial physicians seen]**
   3. What, if any, obstacles do HCPs typically face while making a OI diagnosis?
6. What are the tests, evaluations, and assessments performed to diagnose a patient with OI?
   1. What proportion of patients are diagnosed on the basis of clinical signs and symptoms only? What proportion also undergo:
      1. Radiographic imaging?
      2. Biochemical testing?
      3. Genetic testing? [**Coverage in different geographies]**
   2. In the absence of other signs and symptoms, how many simultaneous or consecutive fractures typically prompt assessment of OI?
   3. How often have you seen parents request genetic testing prenatally for OI? **[Probe on circumstances that lead to prenatal genetic testing and the effect a positive result for OI may have]**
   4. Which specialties are typically involved in the diagnosis of OI?
      1. To which specialties are patients typically referred first?
      2. How many physicians has a patient typically seen by the time a diagnosis is confirmed?
      3. What role does each HCP play? **[Probe on analyzing specific signs and symptoms (e.g., blue sclera, deformities, developmental delay, etc.)]**
7. Is OI ever misdiagnosed? **[If so]:**
   1. Which condition is OI most often misdiagnosed as?
   2. What are the primary reasons that OI is misdiagnosed as other diseases or causes? **[Probe on suspected abuse as basis for fractures]**
   3. Which physician specialties/site of care are most often responsible for misdiagnosis?
   4. If OI is misdiagnosed, how long does it typically take to realize the misdiagnosis and re-diagnose as OI?
   5. What are the consequences of misdiagnosis/delayed diagnosis?
8. How could the process of patient diagnosis be improved?
   1. What are the key pain points? How could they be alleviated?
   2. Which diagnostic interventions are reimbursed in your country?
   3. Is there anything that could be done to support better or faster diagnosis?
9. What is the typical course of the disease in OI patients?
   1. What primary features of the disease manifest with age? **[Probe on hearing loss, scoliosis, stunted growth, etc.]**
   2. Do certain OI subtypes progress differently than others?
   3. How is disease progression measured? **[Probe on fracture rate vs. BMD vs. other]**
10. What are the most common comorbidities or sequelae that you see among your patients?
    1. How do they differ by age, severity, and subtype?
    2. What are the most severe comorbidities that require treatment? **[Probe on basilar impression, cardiopulmonary complications]**
       1. What proportion of OI patients develop these comorbidities?
       2. What additional specialties are typically involved in the management and treatment of these comorbidities?
    3. Beyond physical comorbidities, what is the psychosocial impact of OI on patients and caregivers?
       1. What proportion of OI patients experience psychosocial effects?
       2. What additional healthcare professionals are typically involved in helping patients and caregivers cope with the psychosocial impact of the disease?
11. What is the referral pathway that brings OI patient(s) to you? **[Probe on the number of HCPs seen prior to you, type of specialists seen, where these specialists are located, etc.]**
    1. Is there a well-established referral pattern that many of your patients go through or is there variance in the referral paths?
       1. If patients go through an established referral pattern, how was this network formed? **[Probe on who started it, is it location-based, reputation-based, etc.]**
       2. How are appropriate treating physicians identified for OI? **[Probe on role of patient advocacy groups]**
    2. Are there any stages or hurdles in the referral pathway where patients are often held up or “lost”? Why?
    3. Do you ever see patients who suspect their diagnosis after their own research (e.g., online) without having been referred from other HCPs?
    4. Do you ever have patients come to you from outside the country for treatment/care? Do you ever refer patients out of country for treatment/care? If so, what countries and what kinds of treatments/care?
12. **CURRENT TREATMENT AND MANAGEMENT**
13. Is there a typical treatment team for the management of OI? Which specialties are involved, and what are their roles?
    1. Is there any one specialty or provider type that serves as the “medical home” for OI patients?
       1. Is this provider typically responsible for coordinating care with other specialties? If not, whose responsibility is care coordination? **[Probe on designated non-physician care coordinator vs. patient / caregiver]**
    2. Beyond physician specialists, what other healthcare professionals are involved in the management of OI and its sequelae? **[Probe on physical therapy, social work, psychology, etc.]**
    3. Are there recognized Centers of Excellence or designed OI clinics where management of OI is concentrated?
       1. How many such centers are there in your country?
       2. How common is it for patients to travel long distances to receive care at such centers? **[Probe on frequency of visits]**
       3. What parts of patient treatment and management tend to occur near/in the patients’ hometown vs. in specialized centers?
    4. In the absence of specialist care, are PCPs/GPs aware of the basic treatment principles?
       1. What aspects of patient care tend to be missed or overlooked when patients lack access to specialist care?
14. What pharmacological interventions are typically used in the management of OI?
    1. How would you describe your primary goals when treating OI pharmacologically?
       1. What signs and symptoms are you most focused on managing?
    2. What is considered the standard of care for pharmacological intervention?
       1. **[If bisphosphonates]** Which bisphosphonates are most commonly used? Through what route of administration and with what frequency? **[Type used, peds vs adults?]**
       2. Is there a specific threshold (e.g., number or rate of fractures) that prompts pharmacological treatment?
       3. What is the typical duration of therapy? **[Probe on age / developmental stage when treatment with bisphosphonates is discontinued]**
       4. What are the main strengths of the standard of care? Main weaknesses?
       5. What proportion of patients still experience fractures while receiving the standard of care?
       6. How, if at all, does the treatment approach differ by patient age, subtype, or disease severity? **[Probe on evaluation criteria used to define severity]**
       7. Do any particular types of patients respond better or worse to the standard of care than others?
    3. For patients with inadequate response, what is the next step or treatment approach?
       1. At what point, if at all, do you feel that you have exhausted treatment options? Is it at a certain age, is it after a sequence of therapies?
    4. Under what circumstances are pharmacological interventions outside of the standard of care used? **[Probe on frequency of use of romosozumab, denosumab, and teriparatide]**
    5. Which physicians or specialties tend to prescribe pharmacological interventions for OI?
       1. Is this physician typically considered the primary decision-maker for the patient’s care?
    6. Do patients ever experience challenges affording or obtaining insurance coverage for these pharmacological interventions? If so, under what circumstances?
15. What non-pharmacological interventions (e.g., surgeries, bracing, rehabilitation) are typically used in the management of OI?
    1. What are the most common surgeries that OI patients receive?
       1. Which surgical specialties are typically involved? **[Probe on orthopedic vs. other surgeons (e.g., neurosurgery vs. orthodontics / dental surgery)]**
       2. What proportion of patients undergo at least one surgery related to OI?
       3. When do OI patients typically have their first surgery? How many surgeries are common in a patients’ lifetime?
    2. What are the most common medical equipment or aids used by OI patients?
    3. How many of your patients undergo rehabilitative interventions (e.g., physical or occupational therapy) to treat OI?
       1. For what reasons do patients require rehabilitation?
       2. What rehabilitative services are most commonly accessed?
    4. How many of your patients access some form of psychosocial assistance or care?
       1. For what reasons do patients seek out psychosocial assistance?
    5. Do patients ever experience challenges affording or obtaining insurance coverage for these non-pharmacological interventions?
16. What are the factors that govern how and when the treatment and management of OI should change or evolve for a patient?
    1. How do you typically manage patients transitioning into adulthood?
       1. Do patients maintain their “medical home” or are they referred out to other specialties or centers of excellence?
       2. Are providers and the larger care team primarily responsible for transitioning patients or must patients take the lead? **[Probe on role of patient advocacy groups in coordinating transition of care]**
    2. What expectations do patients have in terms of ongoing care following the transition to adulthood?
       1. Do patients remain open to follow up? **[Probe on what causes them to be lost to follow-up]**
17. What do you see as the biggest challenges or pain points in the treatment and management of OI patients? Probe on:
    1. Lack of indicated treatments, consistency of outcomes, pain management, long-term impact of treatment
    2. Management of comorbidities, sequelae, and psychosocial needs
    3. Impact of transition to adulthood on consistency and quality of care
18. **UNMET NEEDS IN OI**
19. On a scale from 1 to 5, where 1 is no unmet need and 5 is significant unmet need, how would you rate the unmet need for treatment in OI?
    1. What would you consider to be the biggest unmet needs with OI?
    2. What are the key unmet needs at each point in the patient journey? **[Probe on diagnosis and referral pathways, lack of available therapies, difficulty coordinating care, and transition between points of care]**
20. Are there specific unmet needs that are a higher burden on the patient / caregiver?
    1. How would you rank these unmet needs based on level of burden for the patient?
    2. How do these unmet needs impact the quality of life of the patients and caregivers? **[Probe on the social, psychological, and economical impact on QoL, level of anxiety over fractures]**
    3. What is the impact of recurring fractures on the overall long-term health of these patients?
21. How satisfied are you with the current options available to treat OI?
22. Are there any clinical impacts of the disease for which you lack effective treatment options?
23. How much does regulatory approval specifically for OI impact your ability or willingness to prescribe pharmacological therapies? **[Probe on relationship between formal OI indication and access / reimbursement]**
24. Are you aware of any new therapies in clinical development for OI patients? If so, which one(s)?
25. **Conclusion**
26. Is there anything that we have not yet discussed during today’s discussion related to the presentation, diagnosis, and management of OI patients that would be important to discuss?
